# The Major Symptoms of Hysteria

**Published:** 1908-09

**Authors:** 


**The Major Symptoms of Hysteria.** By PIERRE JANET, M.D. Pp. 345. London: Macmillan & Co. Ltd. 1907.

These lectures were delivered by Professor Janet at the inauguration of the new buildings of the medical school of Harvard University at the end of 1906. The lectures are so well expressed and arranged, and at the same time treat the subject in such an interesting manner, that one gains pleasure as well as profit from reading them. Those who are not already acquainted with the author's views should procure this book, which presents them most lucidly, and at moderate length. Professor Janet's work has done much to elucidate hysteria by a process of analysis of the psychical phenomena which lie at its root. We may remind our readers that according to his view these phenomena comprise the occurrence of certain somnambulistic states—that is, of states which may roughly be called "absent-mindedness;" of dissociation of consciousness, extending in its fully-developed form to dual personalities; of limitation, or restriction of the field or range of conscious thought and action; and, lastly, of a lowering of the mental level. We do not suppose for a moment that the last word has been said about hysteria, but no one can read this book without learning something more about the disease, and the modern attempts to explain its nature and origin. Many writers, when they deal with hysteria, either at once become obscure, or make no attempt to inquire into its real nature. This does not apply to Professor Janet, who remains admirably clear throughout, even in the most difficult parts of his subject, so that one can never mistake his meaning. We feel sure that this is a book which, once taken up, will not be left unfinished.

